# Vascular endothelial growth factor promotes atrial arrhythmias by inducing acute intercalated disk remodeling

**DOI:** 10.1038/s41598-020-77562-5

**Published:** 2020-11-24

**Authors:** Louisa Mezache, Heather L. Struckman, Amara Greer-Short, Stephen Baine, Sándor Györke, Przemysław B. Radwański, Thomas J. Hund, Rengasayee Veeraraghavan

**Affiliations:** 1 Department of Biomedical Engineering, College of Engineering, The Ohio State University, 460 Medical Center Dr., Rm 415A, IBMR, Columbus, OH 43210 USA; 2 The Frick Center for Heart Failure and Arrhythmia, Dorothy M. Davis Heart and Lung Research Institute, College of Medicine, The Ohio State University Wexner Medical Center, Columbus, OH USA; 3 Department of Physiology and Cell Biology, College of Medicine, The Ohio State University, Columbus, OH USA; 4 Division of Pharmacy Practice and Sciences, College of Pharmacy, The Ohio State University, Columbus, OH USA

**Keywords:** Arrhythmias, Vascular diseases

## Abstract

Atrial fibrillation (AF) is the most common arrhythmia and is associated with inflammation. AF patients have elevated levels of inflammatory cytokines known to promote vascular leak, such as vascular endothelial growth factor A (VEGF). However, the contribution of vascular leak and consequent cardiac edema to the genesis of atrial arrhythmias remains unknown. Previous work suggests that interstitial edema in the heart can acutely promote ventricular arrhythmias by disrupting ventricular myocyte intercalated disk (ID) nanodomains rich in cardiac sodium channels (Na_V_1.5) and slowing cardiac conduction. Interestingly, similar disruption of ID nanodomains has been identified in atrial samples from AF patients. Therefore, we tested the hypothesis that VEGF-induced vascular leak can acutely increase atrial arrhythmia susceptibility by disrupting ID nanodomains and slowing atrial conduction. Treatment of murine hearts with VEGF (30–60 min, at clinically relevant levels) prolonged the electrocardiographic P wave and increased susceptibility to burst pacing-induced atrial arrhythmias. Optical voltage mapping revealed slower atrial conduction following VEGF treatment (10 ± 0.4 cm/s vs. 21 ± 1 cm/s at baseline, *p* < 0.05). Transmission electron microscopy revealed increased intermembrane spacing at ID sites adjacent to gap junctions (GJs; 64 ± 9 nm versus 17 ± 1 nm in controls, *p* < 0.05), as well as sites next to mechanical junctions (MJs; 63 ± 4 nm versus 27 ± 2 nm in controls, *p* < 0.05) in VEGF–treated hearts relative to controls. Importantly, super-resolution microscopy and quantitative image analysis revealed reorganization of Na_V_1.5 away from dense clusters localized near GJs and MJs to a more diffuse distribution throughout the ID. Taken together, these data suggest that VEGF can acutely predispose otherwise normal hearts to atrial arrhythmias by dynamically disrupting Na_V_1.5-rich ID nanodomains and slowing atrial conduction. These data highlight inflammation-induced vascular leak as a potential factor in the development and progression of AF.

## Introduction

Atrial fibrillation (AF) is the most common cardiac arrhythmia, affecting 2–3% of the US population^1^. Inflammation, vascular leak, and associated tissue edema are common sequelae of pathologies associated with AF^2–8^, and are emerging as proarrhythmic factors. Inflammatory signaling involving cytokines compromises the vascular barrier function, and increase vascular leak^9^. Specifically, multiple studies in early stage AF patients (lone/paroxysmal AF) report elevated levels of vascular endothelial growth factor A (VEGF; 89–560 pg/ml)^3–6,8^ and VEGF receptor 2, its primary receptor in the vascular endothelium^7^. Likewise, elevated levels of vascular leak-inducing cytokines predict AF recurrence following ablation^10^. Although vascular leak is known to promote adverse remodeling and cardiovascular disease in the chronic condition (days-weeks)^11–13^, its acute (< 4 h) contribution to arrhythmogenesis has yet to be explored. Myocardial edema, a direct consequence of vascular leak, is linked to arrhythmias in multiple pathologies, including AF^14–18^. Likewise, cardiac edema has been linked to AF recurrence following ablation^19,20^. Previous work by us and others, suggests that interstitial edema can acutely (within minutes) elevate arrhythmia susceptibility^21–24^. In these studies, the proarrhythmic impact of edema resulted from disruption of cardiac sodium channel (Na_V_1.5)–rich intercalated disk (ID) nanodomains and consequent slowing of action potential propagation^22–25^. Interestingly, similar disruption of ID nanodomains has been identified in AF patients^26^. Therefore, we hypothesized that VEGF (at clinically-relevant levels) may acutely promote atrial arrhythmias by disrupting ID nanodomains and slowing atrial conduction. We provide structural and functional evidence, from the nanoscale to the in vivo level, demonstrating that this mechanism can promote atrial arrhythmias. We also identify a novel form of tissue remodeling involving the dynamic reorganization of Na_V_1.5 within the ID occurring in the aftermath of acute exposure to VEGF, resulting in the dispersal of channels from dense clusters located within nanodomains.

## Methods

All animal procedures were approved by Institutional Animal Care and Use Committee at The Ohio State University and performed in accordance with the Guide for the Care and Use of Laboratory Animals published by the U.S. National Institutes of Health (NIH Publication No. 85-23, revised 2011).

### Langendorff preparation, tissue collection

Male C57/BL6 mice (30 g, 6–18 weeks) were anesthetized with 5% isoflurane mixed with 100% oxygen (1 l/min). After loss of consciousness, anesthesia was maintained with 3–5% isoflurane mixed with 100% oxygen (1 l/min). Once the animal was stably in a surgical plane of anesthesia, the heart was excised, leading to euthanasia by exsanguination. The isolated hearts were prepared in one of the following three ways:

i) Langendorff preparations: For optical mapping and ex vivo electrocardiography (ECG) studies, hearts were perfused (at 60–80 mm Hg) in a Langendorff configuration with oxygenated, modified Tyrode’s solution (containing, in mM: NaCl 140, KCl 5.4, MgCl_2_ 0.5, CaCl_2_ 1.2, dextrose 5.6, HEPES 10; pH adjusted to 7.4) at 37 °C as previously described^22,25,27–29^.
ii) Cryopreservation: Hearts were embedded in optimal cutting temperature compound and frozen using liquid nitrogen for cryosectioning and fluorescent immunolabeling as in previous studies^22,23,25,30^. These samples were used for light microscopy experiments as described below.
iii) Fixation for Transmission Electron Microscopy (TEM): Atria were dissected and fixed overnight in 2% glutaraldehyde at 4 °C for resin embedding and ultramicrotomy as previously described^22,25^.

For both structural and functional studies, the left atrium was prioritized in order to avoid any influence from pacemaker tissue.

### FITC-dextran extravasation

Langendorff-perfused mouse hearts were perfused for 60 min with Tyrode’s solution with or without VEGF (500 pg/ml) and FITC-dextran (10 mg/ml) was added to the final 10 ml of perfusate. Perfused hearts were then cryopreserved as described above and extravasated FITC-dextran levels assessed by confocal microscopy of cryosections.

### Optical mapping and volume-conducted electrocardiography (ECG)

Optical voltage mapping was performed using the voltage sensitive dye, di-4-ANEPPS (15 µM; ThermoFisher Scientific, Grand Island, NY), as previously described^22,23,29^, in order to quantify conduction velocity. Motion was suppressed by adding blebbistatin (10 µM) to the perfusate. Preparations were excited by 510 nm light and fluorescent signals passed through a 610 nm longpass filter (Newport, Irvine, CA) and recorded at 1000 frames/sec using a MiCAM Ultima-L CMOS camera (SciMedia, Costa Mesa, CA). Activation time was defined as the time of the maximum first derivative of the AP^31^, and activation times were fitted to a parabolic surface^32^. Gradient vectors evaluated along this surface were averaged along the fast axis of propagation (± 15°) to quantify CV. Hearts were paced epicardially from the left atrium at a cycle length of 100 ms with 1 ms current pulses at 1.5 times the pacing threshold for all CV measurements. A volume-conducted ECG was collected concurrently using silver chloride electrodes placed in the bath and digitized at 1 kHz. Atrial arrhythmia inducibility was assessed by 10 s of burst pacing at cycle lengths of 50, 40, and 30 ms as previously described^33,34^.

In subsets of experiments, vascular endothelial growth factor A (VEGF; Sigma SRP4364) was added to the perfusate at 100 (low) and 500 pg/ml (high). These concentrations were selected based on VEGF levels observed in human AF patients (89–560 pg/ml)^3–6,8^. Measurements were made following 30 min of treatment.

### In vivo ECG

Continuous ECG recordings (PL3504 PowerLab 4/35, ADInstruments) were obtained from mice anesthetized with isoflurane (1–1.5%) as previously described^35^. Briefly, after baseline recording (5 min.), animals received either intraperitoneal VEGF (10 or 50 ng/kg; Sigma) or vehicle (PBS). After an additional 20 min, animals were injected intraperitoneally with epinephrine (1.5 mg/kg; Sigma) and caffeine (120 mg/kg; Sigma) challenge and ECG recording continued for 40 min. ECG recordings were analyzed using the LabChart 8 software (ADInstruments).

### Primary antibodies

The following primary antibodies were used for Western immunoblotting and fluorescence microscopy studies:

- Connexin43 (Cx43; rabbit polyclonal; Sigma C6219)
- Connexin40 (Cx40; rabbit polyclonal; ThermoFisher Scientific 36–4900)
- N-cadherin (N-cad; mouse monoclonal; BD Biosciences 610,920)
- Cardiac isoform of the voltage-gated sodium channel (Na_V_1.5; rabbit polyclonal; custom antibody^25^)
- The sodium channel β subunit (β1; rabbit polyclonal; custom antibody^25^)

### Western immunoblotting

Whole cell lysates of mouse hearts frozen using liquid nitrogen were prepared as previously described^25,35,36^. These were electrophoresed on 4–15% TGX Stain-free gels (BioRad, Hercules, CA) before being transferred onto a nitrocellulose membrane. The membranes were probed with primary antibodies against Cx43, Cx40, Na_V_1.5 and β1 as well as mouse monoclonal antibody against GAPDH (loading control; Fitzgerald Industries, Acton, MA), followed by goat anti-rabbit and goat anti-mouse HRP-conjugated secondary antibodies (Promega, Madison, WI). Signals were detected by chemiluminescence using SuperSignal West Femto Extended Duration Substrate (ThermoFisher Scientific, Grand Island, NY), imaged using a Chemidoc MP imager (BioRad, Hercules, CA), and analyzed using Image Lab software (BioRad, Hercules, CA).

### Fluorescent immunolabeling

Immuno-fluorescent labeling of cryosections (5 µm thickness) of fresh-frozen myocardium was performed, as previously described ^22,25,35,37^. Briefly, cryosections were fixed with paraformaldehyde (2%, 5 min at room temperature), permeabilized with Triton X-100 (0.2% in PBS for 15 min at room temperature) and treated with blocking agent (1% BSA, 0.1% triton in PBS for 2 h at room temperature) prior to labeling with primary antibodies (overnight at 4 °C). Samples were then washed in PBS (3 × 5 min in PBS at room temperature) prior to labeling with secondary antibodies.

For confocal microscopy, samples were then labeled with goat anti-mouse and goat anti-rabbit secondary antibodies conjugated to Alexa 405, Alexa 488, Alexa 568 and Alexa 647 were used (1:8000; ThermoFisher Scientific, Grand Island, NY). Simultaneous labeling with two rabbit or mouse primary antibodies was accomplished by direct fluorophore conjugation of primary antibodies (Zenon labeling kits, ThermoFisher Scientific, Grand Island, NY). Samples were then washed in PBS (3 × 5 min in PBS at room temperature) and mounted in ProLong Gold (Invitrogen, Rockford, IL). For STimulated Emission Depletion (STED) microscopy, samples were prepared similar to confocal microscopy but labeled with Alexa 594 and Atto 647 N fluorophores. For STochastic Optical Reconstruction Microscopy (STORM), samples were labeled with Alexa 647 and Biotium CF 568 fluorophores. STORM samples were then washed in PBS (3 × 5 min in PBS at room temperature) and optically cleared using Scale U2 buffer (48 h at 4 °C) prior to imaging^23,25,30^.

### Transmission electron microscopy (TEM)

TEM images of the ID, particularly gap junctions (GJs) and mechanical junctions (MJs), were obtained at 60,000 × magnification on a FEI Tecnai G2 Spirit electron microscope. Intermembrane distance at various ID sites was quantified using ImageJ (NIH, http://rsbweb.nih.gov/ij/), as previously described^22,25^.

### Sub-diffraction confocal imaging (sDCI)

Confocal imaging was performed using an A1R-HD laser scanning confocal microscope equipped with four solid-state lasers (405 nm, 488 nm, 560 nm, 640 nm, 30 mW each), a 63×/1.4 numerical aperture oil immersion objective, two GaAsP detectors, and two high sensitivity photomultiplier tube detectors (Nikon, Melville, NY). Individual fluorophores were imaged sequentially with the excitation wavelength switching at the end of each frame. Images were collected as z-stacks with fluorophores images sequentially (line-wise) to achieve optimal spectral separation. Sub-diffraction structural information (130 nm resolution) was recovered by imaging with a 12.8 µm pinhole (0.3 Airy units) with spatial oversampling (4 × Nyquist sampling) and applying 3D deconvolution, as previously described^38^.

### STimulated emission depletion (STED) microscopy

Samples were imaged using a time-gated STED 3X system (Leica, Buffalo Grove, IL) based on a TCS SP8 laser scanning confocal microscope and equipped with STED modules, a pulsed white-light laser (470–670 nm; 80 MHz pulse rate), a Plan Apochromat STED WHITE 100×/1.4 numerical aperture oil immersion objective, HyD hybrid detectors, and three STED depletion lasers (775 nm, 660 nm, 592 nm). Depletion beam was applied in the classical vortex donut configuration to achieve the best lateral resolution (25 nm) as well as in a z-donut configuration to achieve the best axial resolution (50 nm). Time gating of light collection (1.5–3.5 ns following each laser pulse) was also applied to aid in achieving optimal resolution. Images were collected as z-stacks with fluorophores images sequentially (line-wise) and subjected to 3D deconvolution. These images were analyzed using object-based segmentation in 3D (OBS3D), as previously described^22,23^.

### Single molecule localization

STORM imaging was performed using a Vutara 352 microscope (Bruker Nano Surfaces, Middleton, WI) equipped with biplane 3D detection, and fast sCMOS imaging achieving 20 nm lateral and 50 nm axial resolution, as previously described ^25,30,36,39^. Individual fluorophore molecules were localized with a precision of 10 nm. The two color channels were precisely registered using localized positions of several TetraSpeck Fluorescent Microspheres (ThermoFisher Scientific, Carlsbad, CA) scattered throughout the field of view, with the procedure being repeated at the start of each imaging session. Protein clustering and spatial organization were quantitatively assessed from single molecule localization data using STORM-RLA, a machine learning-based cluster analysis approach, as previously described^30^.

### Statistical analysis

Treatments were applied in unblinded fashion for all studies. All data which passed the Shaprio-Wilk test for normality were treated as follows. The Wilcoxon signed rank test or a single factor ANOVA was used for single comparisons. For multiple comparisons, the Šidák correction was applied. Fisher’s exact test was used to test differences in nominal data. For non-normal data, a Friedman rank sum test or Kruskal–Wallis 1-way analysis of variance for paired and unpaired data was applied. A *p* < 0.05 was considered statistically significant. All values are reported as mean ± standard error unless otherwise noted. To ensure unbiased results, all image analyses were conducted using automated batch processing algorithms.

## Results

Multiple studies in early stage AF patients (lone/paroxysmal AF) report elevated levels of VEGF (89–560 pg/ml)^3–6,8^ and VEGF receptor 2^7^. In order to assess the acute impact of VEGF on AF susceptibility, we assessed the structural and electrophysiological impacts of treating Langendorff-perfused WT mouse hearts with clinically relevant levels of VEGF (low: 100 pg/ml and high: 500 pg/ml) for 30 min. VEGF-induced vascular leak was first confirmed by extravasation of FITC-dextran from cryosections of VEGF-treated (500 pg/ml) and vehicle control hearts. Levels of FITC-dextran extravasated into VEGF-treated (500 pg/ml) hearts was doubled relative to vehicle controls (201 ± 7% vs. 100 ± 9%, *p* < 0.05, n = 3 hearts/group; Supplementary Fig. 1). These data are consistent with acute enhancement of vascular leak by VEGF.

### Atrial conduction is slowed following acute VEGF treatment

**Figure 1:**
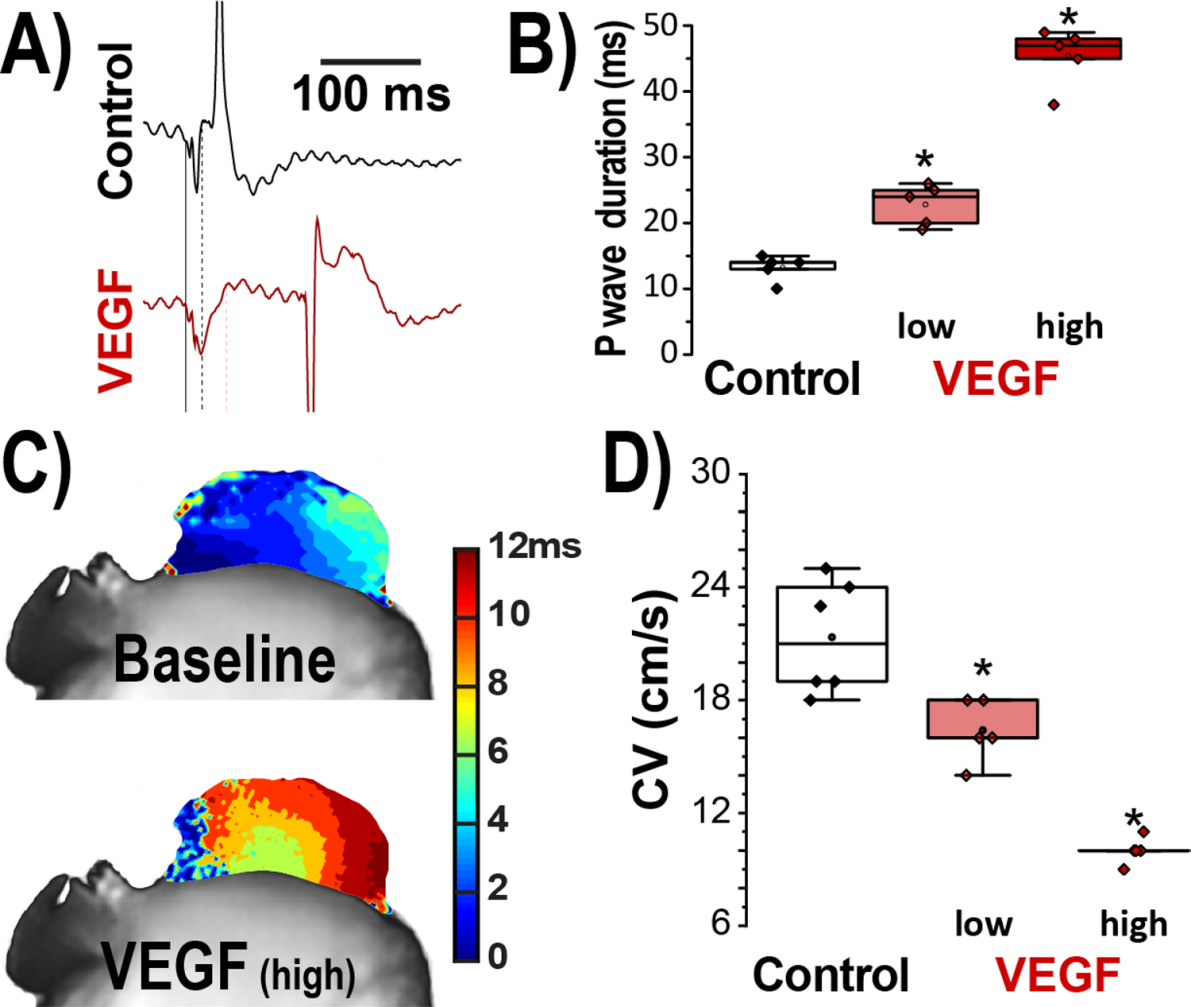
Acute effects of VEGF on atrial conduction. (**A**) Representative volume-conducted ECGs. (**B**) Summary plots of P wave duration (n = 5/group; *p < 0.05 vs. control). (**C**) Representative isochrone maps of left atrial activation. (**D**) Summary plots of CV (n = 5/group; *p < 0.05 vs. control).

To examine the functional impacts of VEGF-induced ID remodeling, volume-conducted electrocardiograms (ECG) were recorded from Langendorff-perfused mouse hearts (Fig. 1). Significant P-wave prolongation was observed following 30 min of VEGF perfusion compared to control (Fig. 1A,B). VEGF exerted similar effects on P-wave duration in vivo (Supplementary Fig. 2). These data point to possible slowing of atrial conduction following VEGF treatment. Next, we directly assessed atrial conduction velocity using optical voltage mapping. Representative optical isochrone maps of activation in Fig. 1C demonstrate increased conduction delay in VEGF treated hearts compared to untreated controls. Overall, VEGF significantly and dose-dependently decreased atrial conduction velocity (Fig. 1D).

### VEGF-treated hearts are susceptible to atrial arrhythmias

**Figure 2:**
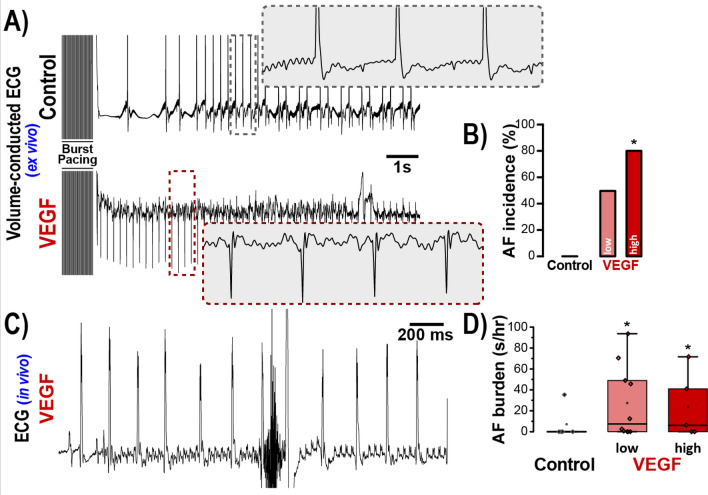
Acute impact of VEGF on atrial arrhythmia susceptibility. (**A**) Representative volume-conducted ECGs show response to burst pacing. (**B**) Incidence of atrial arrhythmias following burst pacing (n = 5/group, * *p* < 0.05 vs. control). (**C**) Representative in vivo surface ECG illustrates atrial arrhythmia observed in a VEGF-treated mouse following caffeine + epinephrine challenge. (**D**) Total atrial arrhythmia burden under caffeine + epinephrine challenge quantified as seconds of arrhythmia per hour of observation (n = 10/group, *p < 0.05 vs. control).

Conduction slowing is a well-established substrate for cardiac arrhythmias in general^40–42^, and AF in particular^43,44^. Therefore, we assessed the acute effects of VEGF-induced conduction slowing on AF risk. A representative volume-conducted ECG trace in Fig. 2A (top) illustrates resumption of sinus rhythm following atrial burst pacing. In contrast, an atrial arrhythmia is apparent on the trace from a VEGF-treated heart (Fig. 2A, bottom). Overall, VEGF increased the incidence of burst pacing-induced atrial arrhythmias in dose-dependent fashion (Fig. 2A,B; Supplementary Fig. 3).

Next, we assessed the acute impact of VEGF on atrial arrhythmia risk in vivo. Promotion of arrhythmic triggers via caffeine and epinephrine challenge elicited atrial arrhythmias in VEGF-treated mice but not in untreated controls (Fig. 2C,D; Supplementary Fig. 4). Taken together, these data suggest that conduction slowing increases the risk of atrial arrhythmias.

### VEGF does not acutely alter expression of key ID proteins

In order to determine the structural basis of VEGF-induced atrial arrhythmias, we assessed the expression of key ID proteins. Western immunoblotting revealed no significant difference in the levels of Na^+^ channel subunits (Na_V_1.5, β1), the gap junction protein Cx43, or the mechanical junction protein, N-cad between VEGF-treated (high dose) hearts and untreated controls (Supplementary Fig. 5). Expression of the gap junction protein Cx40 was slightly elevated in VEGF-treated hearts. Increased Cx40 expression could enhance GJ coupling, although the small change observed is unlikely to have appreciable functional impact. In any case, changes in ID protein expression cannot explain VEGF-induced conduction slowing and proarrhythmia.

### ID structural remodeling following acute VEGF insult

**Figure 3:**
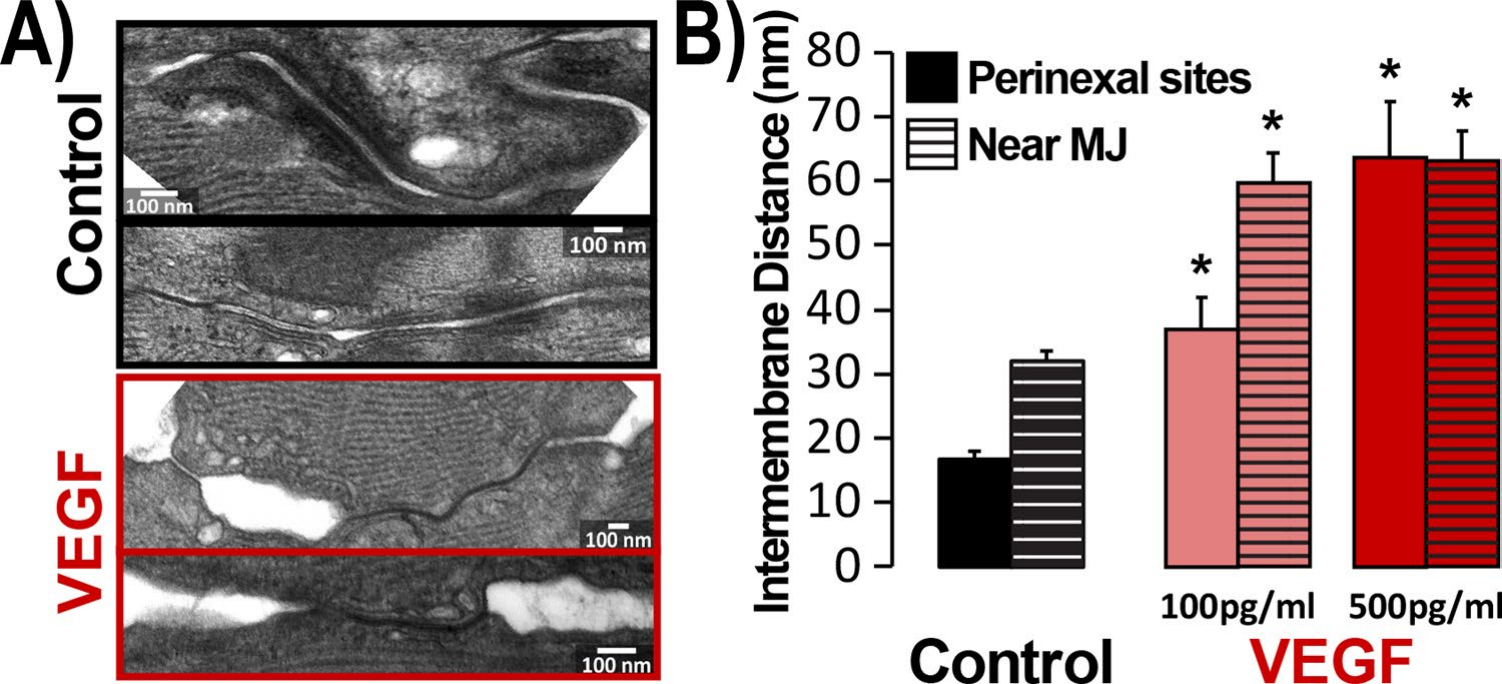
VEGF effects on ID ultrastructure. (**A**) Representative TEM images of IDs. (**B**) Summary plots of intermembrane distance at GJ-adjacent perinexal sites (solid bars) and MJ-adjacent (striped bars) ID sites (> 100 measurements/group/location from n = 3 hearts/group, *p < 0.05 vs. control).

Previous studies link cardiac interstitial edema to ultrastructural remodeling within the ID, specifically, increased intermembrane distance near GJ. Similar changes have also been reported in AF patients^26^. Therefore, we performed transmission electron microscopy (TEM) to assess the acute effects of VEGF on ID structure. Representative TEM images show narrow intermembrane spacing at GJ- and MJ-adjacent sites in untreated control hearts, and marked widening at these sites following VEGF treatment (Fig. 3A). Overall, both low and high doses of VEGF significantly increased intermembrane distances at GJ- and MJ-adjacent sites compared to untreated controls (Fig. 3B). The swelling occurred in dose-dependent fashion at GJ-adjacent perinexi but not near MJ.

### ID proteins undergo reorganization following acute VEGF treatment

**Figure 4:**
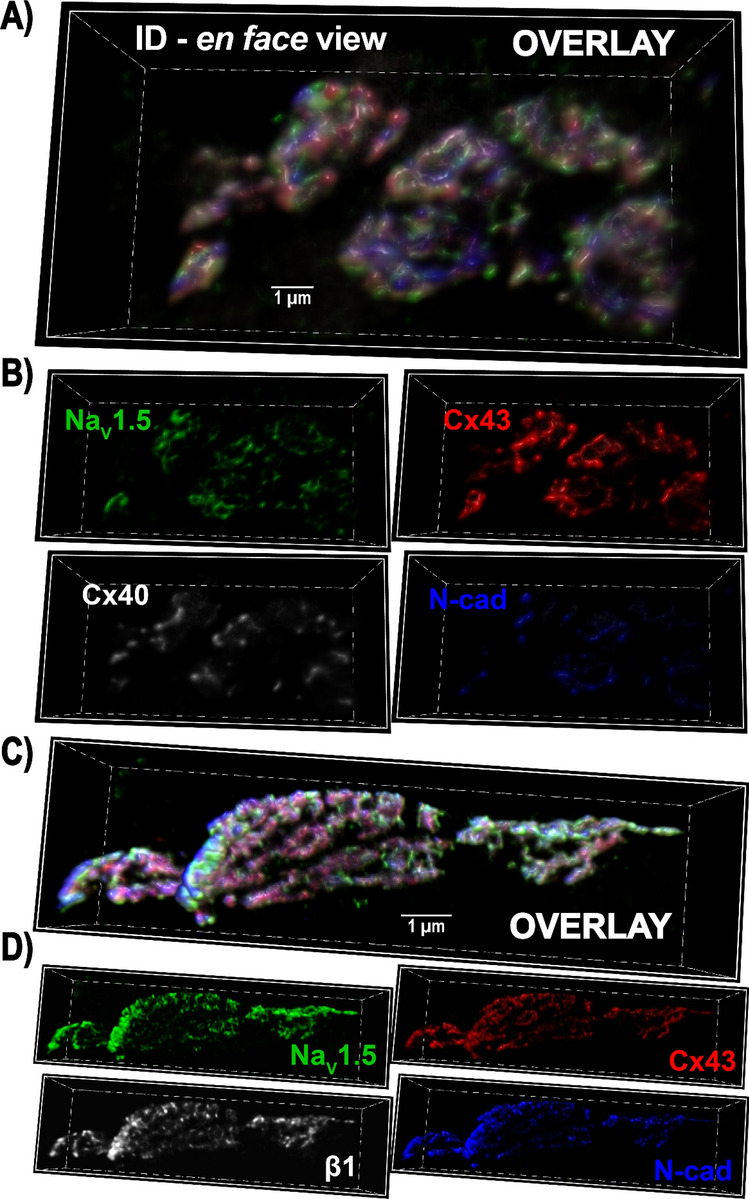
sDCI imaging of IDs. Representative 3D sDCI images of *en face* IDs from murine atria immunolabeled for (A, B) Na_V_1.5, Cx40, Cx43, and N-cad, and (C, D) Na_V_1.5, β1, Cx43, and N-cad.

Next, we performed super-resolution microscopy studies to assess the effects VEGF on ID molecular organization. As a first step, we used sDC imaging (130 nm resolution) to examine the overall layout of key proteins within the murine atrial ID. Although lacking the resolution of other super-resolution imaging methods such as STED and STORM, sDCI offers greater capability for multicolor imaging. Therefore, we used sDCI to examine the organization of sodium channel α (NaV1.5) and β (β1) subunits relative to GJ (Cx40, Cx43) and MJ (N-cad) proteins (Fig. 4).

Both connexin isoforms predominantly expressed in the atria, Cx40 and Cx43, displayed similar patterns of localization (Fig. 4A,B), suggesting that either isoform could be used as a marker for atrial GJs. N-cad immunosignal was localized to distinct ID regions compared to Cx40, Cx43, with very little co-localization. These results are consistent with the enrichment of GJ and MJ within interplicate and plicate ID regions respectively. Representative sDCI images (Fig. 4C,D) illustrate an ID in *en face* orientation from a murine atrial section labeled for Na_V_1.5, β1, Cx43 and N-cad. Na_V_1.5 and β1 were distributed extensively throughout the ID.

**Figure 5:**
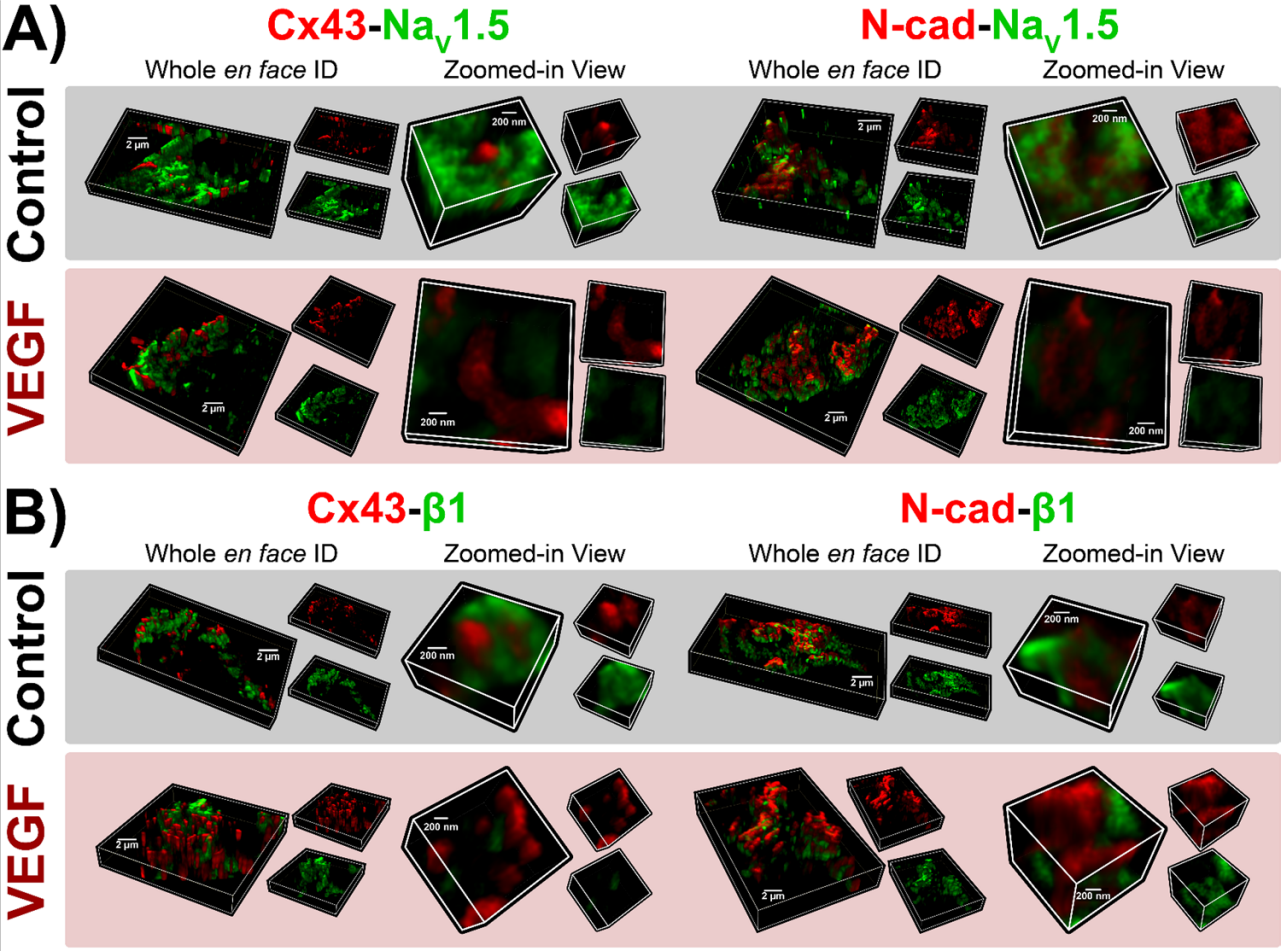
STED imaging of atrial IDs. Representative 3D STED images of *en face* IDs from VEGF-treated and control murine atria immunolabeled for (**A**) Na_V_1.5 and (**B**) β1 along with Cx43 and N-cad.

**Figure 6:**
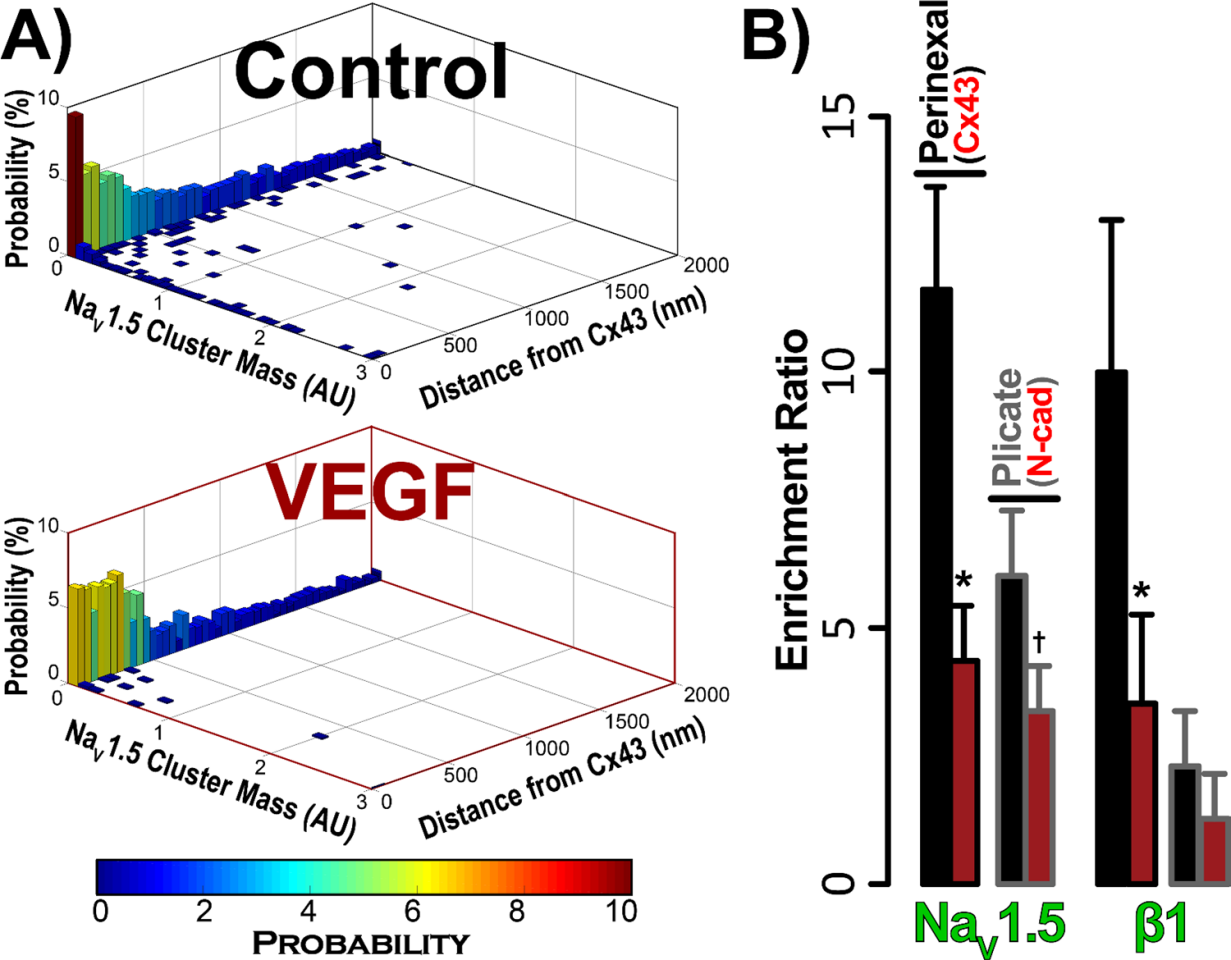
OBS3D analysis of STED images. (**A**) Bivariate histograms of Na_V_1.5 cluster mass (normalized intensity summed over the cluster) as a function of distance from Cx43 clusters. These provide representative examples of intermediate steps in image analysis involved in assessing enrichment ratios, calculated as the ratio of Na_V_1.5/β1 immunosignal cluster mass (volume x normalized intensity) at sites near (< 100 nm away) Cx43 (GJ) and N-cad (MJ) clusters vs. the signal cluster mass at other ID sites. (**B**) Summary plots of enrichment ratio (n = 3 hearts/group, 3 images/heart; *p < 0.05 vs. control).

Having established the overall layout of Na^+^ channel components within the atrial ID, we switched to higher resolution techniques to assess the effects of VEGF-induced vascular leak on their localization. Three dimensional *en face* views of IDs from control hearts obtained by STED microscopy (25 nm resolution) reveal extensive clustering of Na_V_1.5 throughout the ID, particularly in close proximity to Cx43 clusters and at N-cad-rich sites (Fig. 5A, top). In VEGF-treated hearts, Na_V_1.5 clusters appeared fragmented, were located further from Cx43 clusters, and co-distributed less with N-cad (Fig. 5A, bottom). Similar to Na_V_1.5, β1 was also organized into clusters in control hearts, and was found in close proximity to Cx43 clusters (Fig. 5B, top). However, unlike Na_V_1.5, β1 displayed very little co-distribution with N-cad. In VEGF-treated hearts, β1 clusters appeared more diffuse and were distributed farther away from Cx43 clusters (Fig. 5B, bottom). Quantitative analysis by object-based segmentation was used to calculate Na_V_1.5 and β1 signal enrichment ratio, defined as the ratio of Na_V_1.5 / β1 immunosignal cluster mass (volume x normalized intensity) at sites near (< 100 nm away) Cx43 and N-cad vs. the signal cluster mass at other ID sites. Overall, we observed significant enrichment of Na_V_1.5 immunosignal near (< 100 nm) Cx43 and N-cad, and β1 near Cx43 in control hearts (Fig. 6). VEGF-treatment significantly decreased Na_V_1.5 and β1 enrichment ratio near Cx43, while Na_V_1.5 also trended towards a decrease at N-cad-rich sites. These results suggest that VEGF-induced vascular leak induces acute nanoscale reorganization of Na_V_1.5 and β1 within the ID.

**Figure 7:**
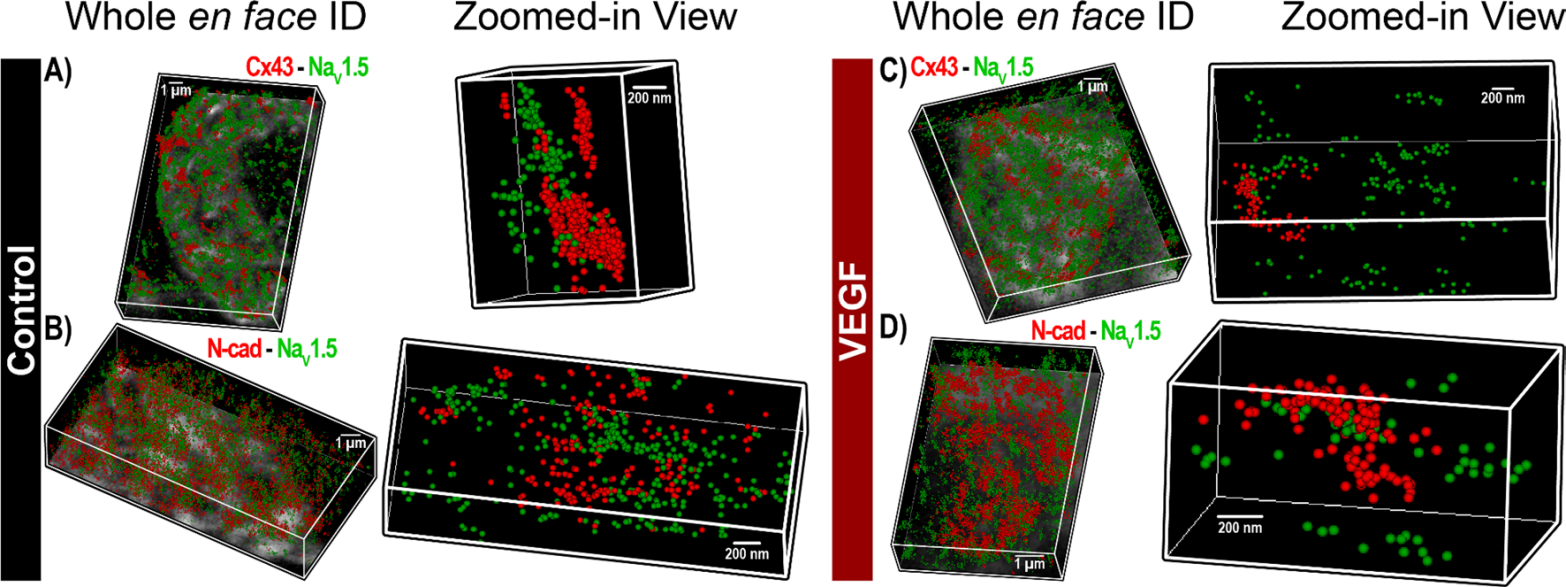
STORM imaging of atrial IDs—Na_V_1.5. Representative 3D STORM images of *en face* IDs immunolabeled for Na_V_1.5 along with Cx43 and N-cad from (**A, B**) control and (**C, D**) VEGF-treated murine atria. STORM data are rendered as point clouds with each localized molecule represented as a 50 nm sphere. Although 20 nm resolution was achieved, the 50 nm size was chosen for rendering to guarantee visibility in print.

**Figure 8:**
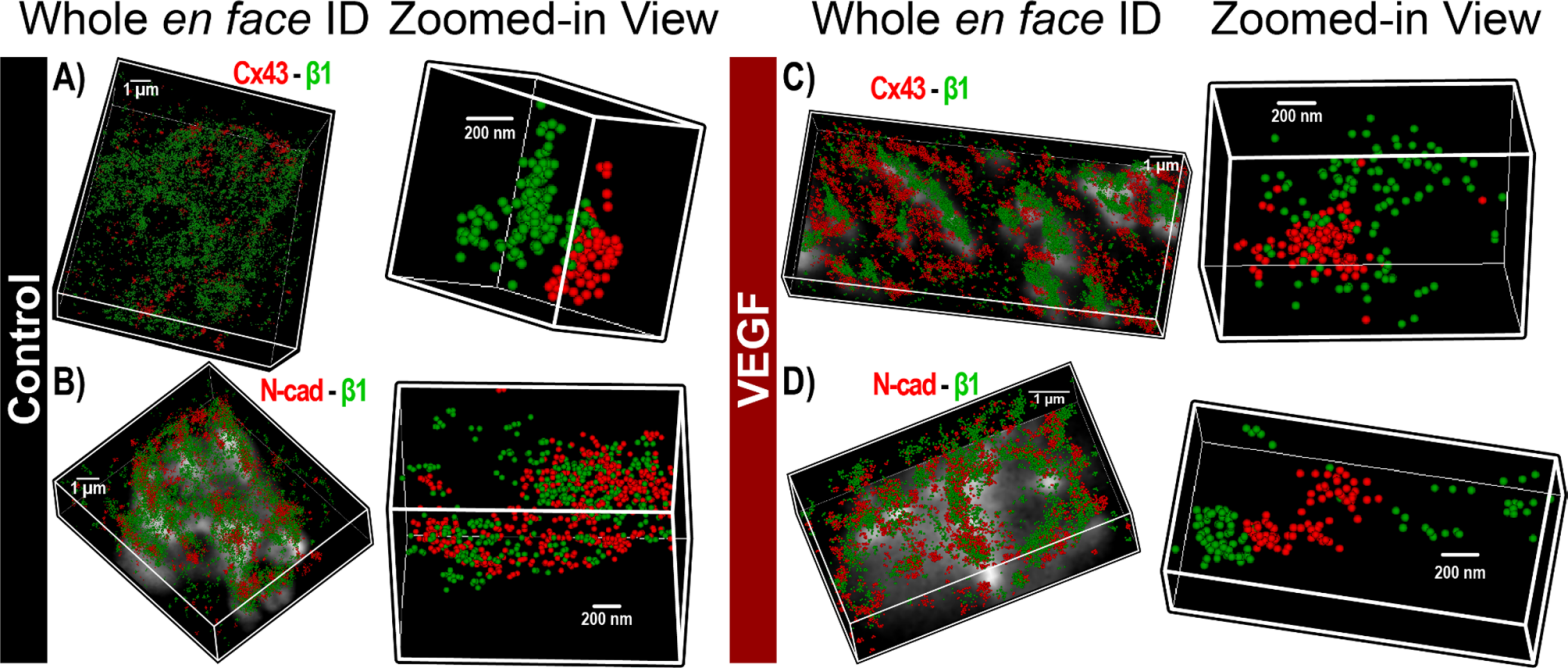
STORM imaging of atrial IDs—β1. Representative 3D STORM images of *en face* IDs immunolabeled for β1 along with Cx43 and N-cad from (**A**, **B**) control and (**C**, **D**) VEGF-treated murine atria.

**Figure 9:**
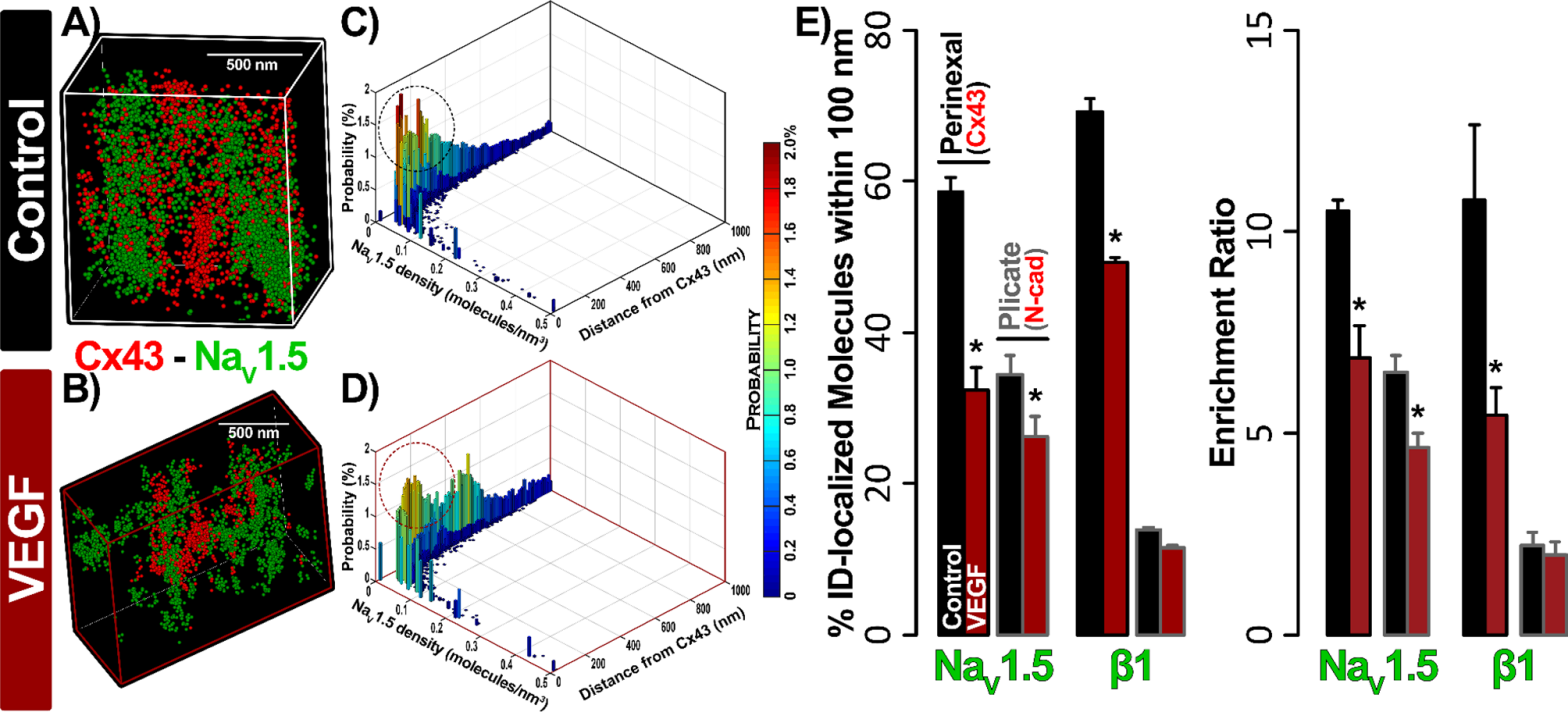
STORM-RLA analysis of Na_V_1.5, β1 localization. Representative 3D STORM images of a Cx43 cluster and associated Na_V_1.5 clusters from (**A**) control and (**B**) VEGF-treated murine atria. (**C, D**) Bivariate histograms of Na_V_1.5 cluster density as a function of distance from Cx43 clusters. Dashed circles highlight the decrease in Na_V_1.5 clusters located near Cx43. (**E**) Summary plots of STORM-RLA results. Left: % of ID-localized Na_V_1.5 and β1 located within 100 nm of Cx43 (GJ) and N-cad (MJ) clusters. Right: Enrichment ratio, calculated as the ratio of Na_V_1.5/β1 cluster density within 100 nm of Cx43 (GJ) and N-cad (MJ) clusters to Na_V_1.5/β1 cluster density at other ID sites (n = 3 hearts/group, 10 images/heart; *p < 0.05 vs. control).

Despite its high resolution, STED microscopy still has limited ability to assess protein density. In any fluorescence image, intensity is determined by a combination of the density of fluorescently-labeled proteins and the number of photons emitted by each. In order to obtain orthogonal validation of the STED results and overcome this limitation, we turned to STORM single molecule localization microscopy and STORM-RLA machine learning-based cluster analysis. By localizing individual molecules, STORM offers the unique ability to assess relative differences in protein density between different ID regions. Representative three-dimensional *en face* views of atrial IDs obtained by STORM show dense clusters of Na_V_1.5 occurring in close proximity to Cx43 and within N-cad-rich regions in control hearts (Fig. 7A,B). In VEGF-treated hearts, Na_V_1.5 clusters appeared more diffuse and were shifted away from Cx43 and N-cad clusters (Fig. 7C,D). In contrast, β1 was preferentially localized near Cx43 clusters and throughout N-cad-free ID regions in control hearts (Fig. 8A,B). In VEGF-treated hearts, β1 clusters appeared further from Cx43 clusters (Fig. 8). Close-up views of Cx43 clusters and associated Na_V_1.5 clusters supported these findings (Fig. 9A,B). STORM data were quantitatively analyzed using STORM-RLA to determine the percent of total Na_V_1.5/β1 signal at the ID, which was localized within Cx43-adjacent perinexal sites (≤ 100 nm from Cx43 clusters; Fig. 9C,E) and at N-cad-rich plicate ID sites (Fig. 9D,E). Additionally, signal enrichment ratio, defined as the ratio of Na_V_1.5/β1 molecular density at these sites vs. the density at other ID sites was also calculated. In control hearts, 59 ± 2% of Na_V_1.5 was localized within Cx43-adjacent perinexal sites (enrichment ratio: 10.5 ± 0.3) and 35 ± 2% within N-cad-rich plicate ID sites (enrichment ratio: 6.5 ± 0.4). In contrast, β1 displayed a marked preference for Cx43-adjacent perinexal sites (69 ± 4% of ID-localized β1, enrichment ratio: 10.7 ± 1.9) in comparison to N-cad-rich plicate ID sites (14 ± 3% of ID-localized β1). In VEGF treated hearts, Na_V_1.5 density was significantly reduced at both Cx43-adjacent perinexal sites (32 ± 3% of signal, enrichment ratio: 6.9 ± 0.8) and N-cad-rich plicate ID sites (26 ± 3% of signal, enrichment ratio: 4.6 ± 0.4). Likewise, β1 density was also reduced at Cx43-adjacent perinexal sites (49 ± 3% of signal, enrichment ratio: 5.4 ± 0.7) without significant changes at N-cad-rich plicate ID sites. Overall, the STORM-RLA results indicated dynamic reorganization of ID-localized Na_V_1.5 and β1 following VEGF treatment.

## Discussion

**Figure 10:**
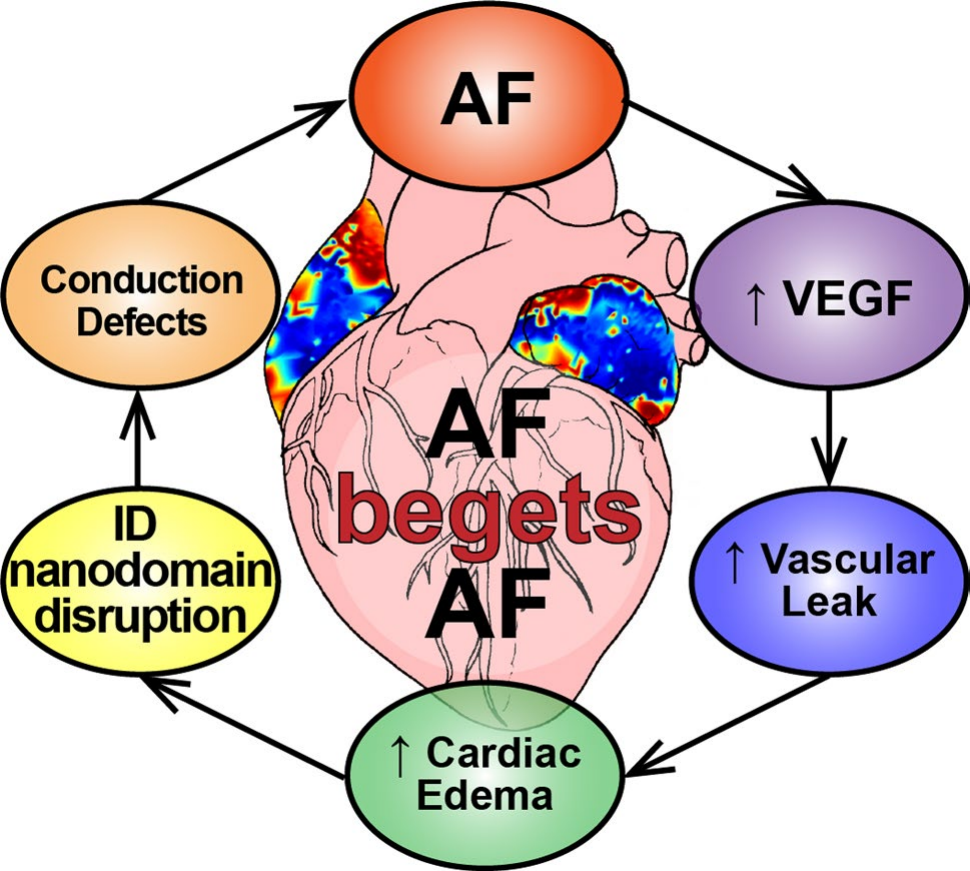
Proposed mechanism for the genesis and progression of AF. Elevated VEGF levels in AF patients increase vascular leak, in turn promoting cardiac edema. The resulting disruption of Na_V_1.5-rich ID nanodomains slows atrial conduction, thereby providing a substrate for further atrial arrhythmias.

Patients with new-onset AF show elevated levels of VEGF^3–6,45^, a cytokine that promotes vascular leak. Indeed, inflammation, vascular leak, and associated tissue edema are common sequelae of AF^2–8^, and are emerging as proarrhythmic factors. In previous studies in the ventricles, myocardial edema acutely (within minutes) disrupted ID nanodomains, slowed conduction, and precipitated arrhythmias^22–24^. Interestingly, patients with AF also evidence swelling of ID nanodomains^26^ and conduction slowing has been linked to AF in human patients^43,44^. However, the mechanism by which tissue edema due to vascular leak precipitates AF is unknown. Therefore, we tested the hypothesis that VEGF may acutely promote atrial arrhythmias by disrupting ID nanodomains and compromising atrial conduction (Fig. 10). Here, we demonstrate that VEGF insult acutely induces ID nanodomain swelling and translocation of sodium channel subunits from these sites, likely generating a substrate for slowed atrial conduction, and atrial arrhythmias.

Cytokines such as VEGF, which induce vascular leak, have been shown to have a multitude of other impacts, including directly reducing the expression of Cx43 in cardiac myocytes^46–51^. In contrast, our Western blots indicated no change in the expression of Cx43 or Na^+^ channel subunits, and a slight increase in Cx40 expression following acute VEGF insult. The apparent divergence of our results from the aforementioned studies may reflect the much longer time courses (> 4 h) involved in those compared to our study (< 1 h). Overall, our data suggest that reduced expression of ID proteins cannot explain the rapid proarrhythmic impact of VEGF in our experiments.

In previous studies, acute interstitial edema induced swelling of the perinexus, a GJ-adjacent ID nanodomain, and brought about conduction slowing and spontaneous arrhythmias within 10 min^22–24^. Likewise, George et al. demonstrated elevated extracellular volume, ID nanodomain swelling, and conduction slowing during acute inflammatory response (90 min of exposure to pathophysiological levels of TNFα)^21^. Consistent with these, our TEM studies identified significant swelling of ID nanodomains (near both GJs and MJs) following VEGF treatment. Taken together, these results suggest that ID nanodomain swelling may contribute to atrial arrhythmias following acute VEGF insult. Notably, the ultrastructural impact of VEGF in our experiments closely corresponds with observations from human AF patients^26^.

A concomitant impact during acute swelling of ID nanodomains, suggested by previous work, is the translocation of sodium channels from these sites^25^. Perinexal swelling was found to decrease local I_Na_ density near GJs, albeit without any change in whole-cell I_Na_ and was sufficient to induce proarrhythmic conduction slowing. These results suggest that the precise localization of sodium channels within the ID may be an important determinant of cardiac electrical propagation. Therefore, we used super-resolution microscopy to test whether VEGF-induced ID remodeling included any reorganization of sodium channel proteins. Overall, STED and STORM both identified Na_V_1.5 enrichment near Cx43 clusters as well as at N-cad-rich sites, consistent with previous reports^22,23,25,30,52^. In contrast, β1 was preferentially localized near Cx43 and predominantly within N-cad-free ID sites, again in keeping with previous data^25^. These data suggest that Na_V_1.5 at N-cad-rich sites may associate with a different β subunit, an idea which merits future investigation. Importantly, both STED and STORM images revealed changes consistent with decreased Na_V_1.5 near GJs and MJs in VEGF-treated hearts relative to controls. Quantitative analysis of STED and STORM data revealed a substantial depletion of Na_V_1.5 from GJ-adjacent perinexal sites, and to a somewhat lesser degree, also from MJ-adjacent sites. Likewise, VEGF treatment also decreased β1 density at GJ-adjacent sites. Overall, these data, along with previously published results^25^, suggest that local I_Na_ density at GJ- and MJ- adjacent sites might be decreased following acute VEGF insult. Taken in the context of our TEM results, these data suggest that intermembrane adhesion within ID nanodomains may play a role in retaining sodium channels at these sites. Inhibition of adhesive interactions may enhance lateral diffusion of ion channels within the membrane, resulting in their dispersal from dense clusters. While further research will be required to uncover the precise mechanism by which nanodomain swelling induces sodium channel translocation, we provide here the first direct demonstration of this dynamic remodeling phenomenon.

Taken together, our light and electron microscopy results identify two forms of dynamic ID remodeling following acute exposure to VEGF: (1) swelling of the extracellular cleft near GJs and MJs, and (2) translocation of Na_V_1.5, wherein dense Na_V_1.5 clusters located near GJs and MJs are redistributed more diffusely. These changes could impair atrial conduction via two, non-mutually exclusive mechanisms: (1) Direct effects on membrane excitability via cooperative activation. The earliest activating Na_V_1.5 channels promote positive feedback activation of further Na_V_1.5 channels, when these channels are tightly clustered, and face a restricted extracellular cleft^53,54^. Na_V_1.5 translocation away from dense clusters into a more diffuse pattern would weaken this effect, and could thereby compromise excitability. (2) Indirect effects on intercellular coupling via ephaptic coupling: When dense Na_V_1.5 clusters from adjacent cells face each other across a narrow (< 30 nm) extracellular cleft, channel activation on one side prompts transient depletion of sodium (positive charge) from the cleft, and subsequent depolarization of the apposed cell’s membrane, activating its Na_V_1.5 channels^55–58^. Both nanodomain swelling and the more diffuse reorganization of Na_V_1.5 would weaken local electrochemical transients within ID nanodomains, and could thereby impair atrial conduction^22,23,25,59–61^. Notably, based on their structural properties, both perinexi and plicate nanodomains would support cooperative activation but only perinexi are predicted to support ephaptic coupling^59,62^. However, since VEGF impacted both locations simultaneously, our results do not delineate the relative contributions of the two mechanisms, or indeed of the two different ID nanodomains. While future work will be required to answer these mechanistic questions, the totality of structural and functional results indicate that VEGF can acutely induce proarrhythmic conduction slowing, and likely does so by disrupting ID nanodomains (Fig. 10).

Our results, identifying acute remodeling of ID nanodomains as an arrhythmia mechanism, have important implications for our broader understanding of arrhythmia substrates. Classically, structural arrhythmia substrates are viewed as being permanent (e.g. an infarct), while functional substrates are thought to be dynamic (e.g. a line of block resulting from repolarization heterogeneities). However, vascular leak-induced edema and consequent nanodomain remodeling, as demonstrated here, may represent a dynamic and transient structural arrhythmic substrate. This may contribute to the intermittent nature of arrhythmias in pathologies such as AF in the early stages. The results presented here also have important implications for the treatment of AF. First, they suggest that therapies which mitigate cytokine-induced vascular leak may be effective in preventing atrial arrhythmias. Second, they suggest that direct targeting of ID nanodomains to prevent swelling and sodium channel translocation could also be an effective antiarrhythmic strategy.

### Limitations

VEGF’s impact on the heart is multi-factorial in nature, involving direct effects on cardiac myocytes as well as effects on non-myocyte cells. These include effects on GJs, which could contribute to conduction slowing^46–51^. Although our Western blot analysis did not identify any decrease in Cx40 or Cx43 expression, functional GJ coupling may have been impacted without altering overall protein expression. However, VEGF’s effects on GJs have been demonstrate to occur over much longer time courses (> 4 h) than those involved in the present study (< 4 h). Intermembrane spacing measured by TEM may have been impacted the effects of glutaraldehyde fixation on tissue^63^. However, such effects would uniformly impact all samples and do not detract from the observation that VEGF increases intermembrane spacing near GJs and MJs. Super-resolution microscopy revealed translocation of Na_V_1.5 from ID nanodomains, occurring in conjunction with increase in intermembrane spacing. While our data link these effects to arrhythmogenic conduction slowing, the inability to separate these two effects experimentally precludes delineation of their relative impacts on conduction. While this merits future investigation using experimental and modeling approaches, our data indicate that remodeling of ID nanodomains secondary to VEGF-induced vascular leak is acutely proarrhythmic.

## Conclusion

In summary, we demonstrate that VEGF, at levels occurring in AF patients, can acutely increase susceptibility to atrial arrhythmias. We provide, to our knowledge, the first evidence that sodium channel clusters at the ID can undergo dynamic reorganization. Importantly, we identify a novel mechanism for atrial arrhythmias, wherein dynamic disruption of ID nanodomains, secondary to VEGF-induced vascular leak, induces proarrhythmic slowing of atrial conduction. This mechanism may contribute to the genesis and progression of AF in the early stages and help explain the link between inflammation and AF. Our work identifies vascular leak and ID nanodomains are potential therapeutic targets for the treatment and prevention of AF in the early stages.

## Supplementary information

Supplementary Information.

## Data Availability

All data generated and analyzed during the present study are available upon request from the corresponding author upon reasonable request.

## References

[1] Zoni-Berisso M, Lercari F, Carazza T, Domenicucci S (2014). Epidemiology of atrial fibrillation: European perspective. Clin. Epidemiol..

[2] Weis SM (2008). Vascular permeability in cardiovascular disease and cancer. Curr. Opin. Hematol..

[3] Li J (2010). Role of inflammation and oxidative stress in atrial fibrillation. Heart Rhythm Off. J. Heart Rhythm Soc..

[4] Ogi H (2010). Is structural remodeling of fibrillated atria the consequence of tissue hypoxia?. Circul. J. Off. J. Jpn. Circul. Soc..

[5] Scridon A (2012). Increased intracardiac vascular endothelial growth factor levels in patients with paroxysmal, but not persistent atrial fibrillation. Europace.

[6] Seko Y, Nishimura H, Takahashi N, Ashida T, Nagai R (2000). Serum levels of vascular endothelial growth factor and transforming growth factor-beta1 in patients with atrial fibrillation undergoing defibrillation therapy. Jpn. Heart J..

[7] Gramley F (2010). Atrial fibrillation is associated with cardiac hypoxia. Cardiovasc. Pathol..

[8] Chung NA (2002). Is the hypercoagulable state in atrial fibrillation mediated by vascular endothelial growth factor?. Stroke.

[9] Sukriti S, Tauseef M, Yazbeck P, Mehta D (2014). Mechanisms regulating endothelial permeability. Pulm. Circ..

[10] Kimura T (2014). Serum inflammation markers predicting successful initial catheter ablation for atrial fibrillation. Heart Lung Circ..

[11] Bertoluci MC (2015). Endothelial dysfunction as a predictor of cardiovascular disease in type 1 diabetes. World J. Diabetes.

[12] de Zeeuw D, Parving HH, Henning RH (2006). Microalbuminuria as an early marker for cardiovascular disease. J. Am. Soc. Nephrol..

[13] Montezano AC (2015). Oxidative stress and human hypertension: vascular mechanisms, biomarkers, and novel therapies. Can. J. Cardiol..

[14] Amano Y (2012). T2-weighted cardiac magnetic resonance imaging of edema in myocardial diseases. Sci. World J..

[15] Boyle A, Maurer MS, Sobotka PA (2007). Myocellular and interstitial edema and circulating volume expansion as a cause of morbidity and mortality in heart failure. J. Cardiac. Fail..

[16] White SK (2015). Remote ischemic conditioning reduces myocardial infarct size and edema in patients with ST-segment elevation myocardial infarction. JACC Cardiovasc. Interv..

[17] Zia MI (2014). Comparison of the frequencies of myocardial edema determined by cardiac magnetic resonance in diabetic versus nondiabetic patients having percutaneous coronary intervention for ST elevation myocardial infarction. Am. J. Cardiol..

[18] Migliore F, Zorzi A, Perazzolo Marra M, Iliceto S, Corrado D (2015). Myocardial edema as a substrate of electrocardiographic abnormalities and life-threatening arrhythmias in reversible ventricular dysfunction of takotsubo cardiomyopathy: imaging evidence, presumed mechanisms, and implications for therapy. Heart Rhythm Off. J. Heart Rhythm Soc..

[19] Neilan TG (2014). Myocardial extracellular volume expansion and the risk of recurrent atrial fibrillation after pulmonary vein isolation. JACC Cardiovasc. Imaging.

[20] Arujuna A (2012). Acute pulmonary vein isolation is achieved by a combination of reversible and irreversible atrial injury after catheter ablation: evidence from magnetic resonance imaging. Circul. Arrhythmia Electrophysiol..

[21] George SA, Calhoun PJ, Gourdie RG, Smyth JW, Poelzing S (2017). TNFalpha modulates cardiac conduction by altering electrical coupling between myocytes. Front. Physiol..

[22] Veeraraghavan R (2015). Sodium channels in the Cx43 gap junction perinexus may constitute a cardiac ephapse: an experimental and modeling study. Pflugers Arch..

[23] Veeraraghavan R, Lin J, Keener JP, Gourdie R, Poelzing S (2016). Potassium channels in the Cx43 gap junction perinexus modulate ephaptic coupling: an experimental and modeling study. Pflugers Arch..

[24] Veeraraghavan R, Salama ME, Poelzing S (2012). Interstitial volume modulates the conduction velocity-gap junction relationship. Am. J. Physiol. Heart Circul. Physiol..

[25] Veeraraghavan R (2018). The adhesion function of the sodium channel beta subunit (beta1) contributes to cardiac action potential propagation. Elife.

[26] Raisch TB (2018). Intercalated disc extracellular nanodomain expansion in patients with atrial fibrillation. Front. Physiol..

[27] Radwanski PB (2015). Neuronal Na+ channel blockade suppresses arrhythmogenic diastolic Ca^2+^ release. Cardiovasc. Res..

[28] Radwanski PB, Veeraraghavan R, Poelzing S (2010). Cytosolic calcium accumulation and delayed repolarization associated with ventricular arrhythmias in a guinea pig model of Andersen-Tawil syndrome. Heart Rhythm Off. J. Heart Rhythm Soc..

[29] Veeraraghavan R, Poelzing S (2008). Mechanisms underlying increased right ventricular conduction sensitivity to flecainide challenge. Cardiovasc. Res..

[30] Veeraraghavan R, Gourdie R (2016). Stochastic optical reconstruction microscopy-based relative localization analysis (STORM-RLA) for quantitative nanoscale assessment of spatial protein organization. Mol. Biol. Cell.

[31] Girouard SD, Laurita KR, Rosenbaum DS (1996). Unique properties of cardiac action potentials recorded with voltage-sensitive dyes. J. Cardiovasc. Electrophysiol..

[32] Bayly PV (1998). Estimation of conduction velocity vector fields from epicardial mapping data. IEEE Trans. Bio-med. Eng..

[33] Greer-Short A (2020). Calmodulin kinase II regulates atrial myocyte late sodium current, calcium handling, and atrial arrhythmia. Heart Rhythm Off. J. Heart Rhythm Soc..

[34] Aschar-Sobbi R (2015). Increased atrial arrhythmia susceptibility induced by intense endurance exercise in mice requires TNFalpha. Nat. Commun..

[35] Koleske M (2018). Tetrodotoxin-sensitive Navs contribute to early and delayed afterdepolarizations in long QT arrhythmia models. J. Gener. Physiol..

[36] Struckman HL (2020). Super-resolution imaging using a novel high-fidelity antibody reveals close association of the neuronal sodium channel NaV1.6 with ryanodine receptors in cardiac muscle. Microsc. Microanal..

[37] Radwański PB (2016). Neuronal Na+ channels are integral components of pro-arrhythmic Na^+^/Ca^2+^ signaling nanodomain that promotes cardiac arrhythmias during β-adrenergic stimulation. JACC Basic Transl. Sci..

[38] Lam F, Cladiere D, Guillaume C, Wassmann K, Bolte S (2017). Super-resolution for everybody: an image processing workflow to obtain high-resolution images with a standard confocal microscope. Methods.

[39] Bonilla IM (2019). Enhancement of cardiac store operated calcium entry (SOCE) within novel intercalated disk microdomains in arrhythmic disease. Sci. Rep..

[40] Kleber AG, Rudy Y (2004). Basic mechanisms of cardiac impulse propagation and associated arrhythmias. Physiol. Rev..

[41] Kleber AG (1999). Discontinuous propagation of the cardiac impulse and arrhythmogenesis. J. Cardiovasc. Electrophysiol..

[42] Radwanski PB, Johnson CN, Gyorke S, Veeraraghavan R (2018). Cardiac arrhythmias as manifestations of nanopathies: an emerging view. Front. Physiol..

[43] Zheng Y, Xia Y, Carlson J, Kongstad O, Yuan S (2017). Atrial average conduction velocity in patients with and without paroxysmal atrial fibrillation. Clin. Physiol. Funct. Imaging.

[44] Lalani GG (2012). Atrial conduction slows immediately before the onset of human atrial fibrillation: a bi-atrial contact mapping study of transitions to atrial fibrillation. J. Am. Coll. Cardiol..

[45] Smorodinova N (2015). Bioptic study of left and right atrial interstitium in cardiac patients with and without atrial fibrillation: interatrial but not rhythm-based differences. PLoS ONE.

[46] Dhein S, Polontchouk L, Salameh A, Haefliger JA (2002). Pharmacological modulation and differential regulation of the cardiac gap junction proteins connexin 43 and connexin 40. Biol. Cell.

[47] Pimentel RC, Yamada KA, Kleber AG, Saffitz JE (2002). Autocrine regulation of myocyte Cx43 expression by VEGF. Circ. Res..

[48] Fernandez-Cobo M, Gingalewski C, Drujan D, De Maio A (1999). Downregulation of connexin 43 gene expression in rat heart during inflammation. The role of tumour necrosis factor. Cytokine.

[49] Herve JC, Dhein S (2006). Pharmacology of cardiovascular gap junctions. Adv. Cardiol..

[50] Salameh A (2004). Chronic regulation of the expression of gap junction proteins connexin40, connexin43, and connexin45 in neonatal rat cardiomyocytes. Eur. J. Pharmacol..

[51] Sawaya SE (2007). Downregulation of connexin40 and increased prevalence of atrial arrhythmias in transgenic mice with cardiac-restricted overexpression of tumor necrosis factor. Am. J. Physiol. Heart Circul. Physiol..

[52] Leo-Macias A (2016). Nanoscale visualization of functional adhesion/excitability nodes at the intercalated disc. Nat. Commun..

[53] Hichri E, Abriel H, Kucera JP (2017). Distribution of cardiac sodium channels in clusters potentiates ephaptic interactions in the intercalated disc. J. Physiol..

[54] Clatot J (2017). Voltage-gated sodium channels assemble and gate as dimers. Nat. Commun..

[55] Veeraraghavan R, Gourdie R, Poelzing S (2014). Mechanisms of cardiac conduction: a history of revisions. Am. J. Physiol. Heart Circul. Physiol..

[56] Veeraraghavan R, Poelzing S, Gourdie RG (2014). Old cogs, new tricks: a scaffolding role for connexin43 and a junctional role for sodium channels?. FEBS Lett..

[57] Veeraraghavan R, Poelzing S, Gourdie RG (2014). Intercellular electrical communication in the heart: a new, active role for the intercalated disk. Cell Commun. Adhes..

[58] Veeraraghavan R, Radwanski PB (2018). Sodium channel clusters: harmonizing the cardiac conduction orchestra. J. Physiol..

[59] Mori Y, Fishman GI, Peskin CS (2008). Ephaptic conduction in a cardiac strand model with 3D electrodiffusion. Proc. Natl. Acad. Sci. U.S.A..

[60] Kucera JP, Rohr S, Rudy Y (2002). Localization of sodium channels in intercalated disks modulates cardiac conduction. Circ. Res..

[61] Lin J, Keener JP (2010). Modeling electrical activity of myocardial cells incorporating the effects of ephaptic coupling. Proc. Natl. Acad. Sci. U.S.A..

[62] Lin J, Keener JP (2013). Ephaptic coupling in cardiac myocytes. IEEE Trans. Bio-med. Eng..

[63] Raisch T, Khan M, Poelzing S (2018). Quantifying intermembrane distances with serial image dilations. J. Vis. Exp. JoVE.

