# Supplementary material for: Vascular endothelial growth factor promotes atrial arrhythmias by inducing acute intercalated disk remodeling
Source: Sci Rep. 2020 Nov 24;10:20463. doi: 10.1038/s41598-020-77562-5 (PMC7687901; doi:10.1038/s41598-020-77562-5)
Supplement: Supplementary file 1 — Supplementary Information. [file 41598_2020_77562_MOESM1_ESM.docx]

**Vascular Endothelial Growth Factor Promotes Atrial Arrhythmias by Inducing Acute Intercalated Disk Remodeling**

**Authors:** Louisa Mezache^1^, Heather L. Struckman^1^, Amara Greer-Short, Ph.D.^2^, Stephen Baine, Ph.D.^2,3^, Sándor Györke, Ph.D.^2,3^, Przemysław B. Radwański, Pharm.D., Ph.D.^2,3,4^, Thomas J Hund, Ph.D.^1,2^, Rengasayee Veeraraghavan, Ph.D.^1,2,3^

**Affiliations:**

*^1^Department of Biomedical Engineering, College of Engineering, The Ohio State University, Columbus, OH, US*

*^2^* *The Frick Center for Heart Failure and Arrhythmia, Dorothy M. Davis Heart and Lung Research Institute, College of Medicine, The Ohio State University Wexner Medical Center, Columbus, OH, US*

*^3^Department of Physiology and Cell Biology, College of Medicine, The Ohio State University, Columbus, OH, US*

*^4^Division of Pharmacy Practice and Sciences, College of Pharmacy, The Ohio State University, Columbus, OH, US*

Short Title: **VEGF promotes AF**

**Corresponding Author:**

**Rengasayee Veeraraghavan, Ph.D.
Assistant Professor**

**Dept. of Biomedical Engineering**

**The Ohio State University**

460 Medical Center Dr., Rm 415A, IBMR

Columbus, Ohio 43210.

TEL: 614 366 2694

email: veeraraghavan.12@osu.edu

**SUPPLEMENTARY FIGURE 1**

**
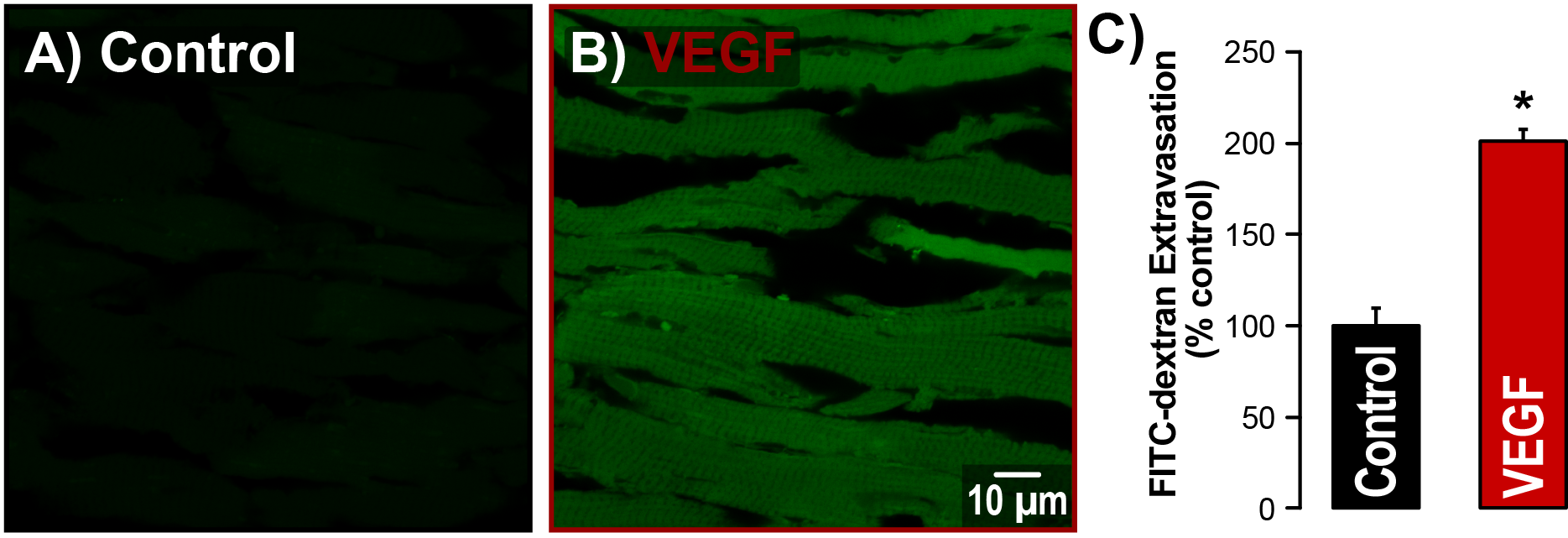
**

**Supplementary Figure 1. VEGF increases vascular leak. A, B)** Representative confocal images and **C)** summary data demonstrate increased extravasation of FITC-dextran in VEGF-treated hearts relative to untreated controls.

**SUPPLEMENTARY FIGURE 2**

**
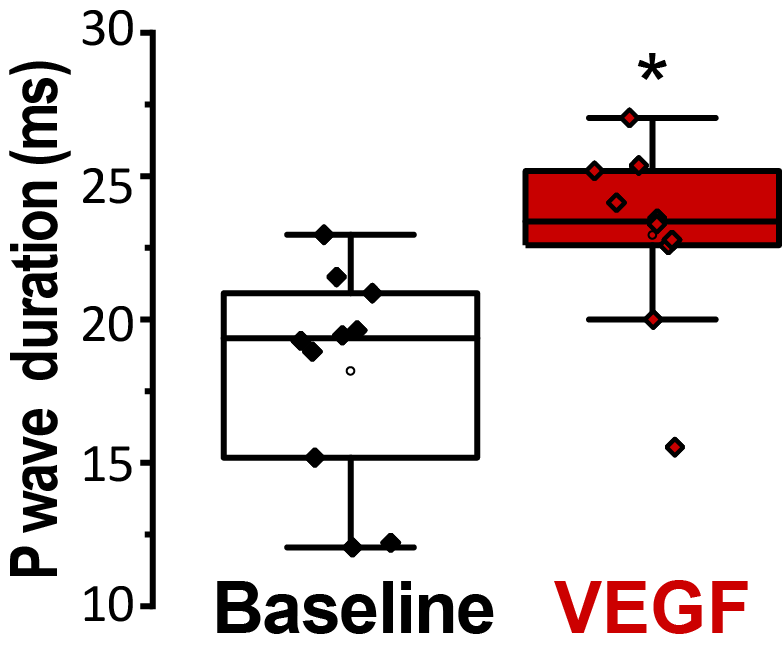
**

**Supplementary Figure 2. VEGF prolongs P-wave.** P wave duration measured from *in vivo* ECG studies in anesthetized mice before and after VEGF (100 ng/l) treatment. (*, p<0.05 vs. pre-VEGF baseline).

**SUPPLEMENTARY FIGURE 3**

**
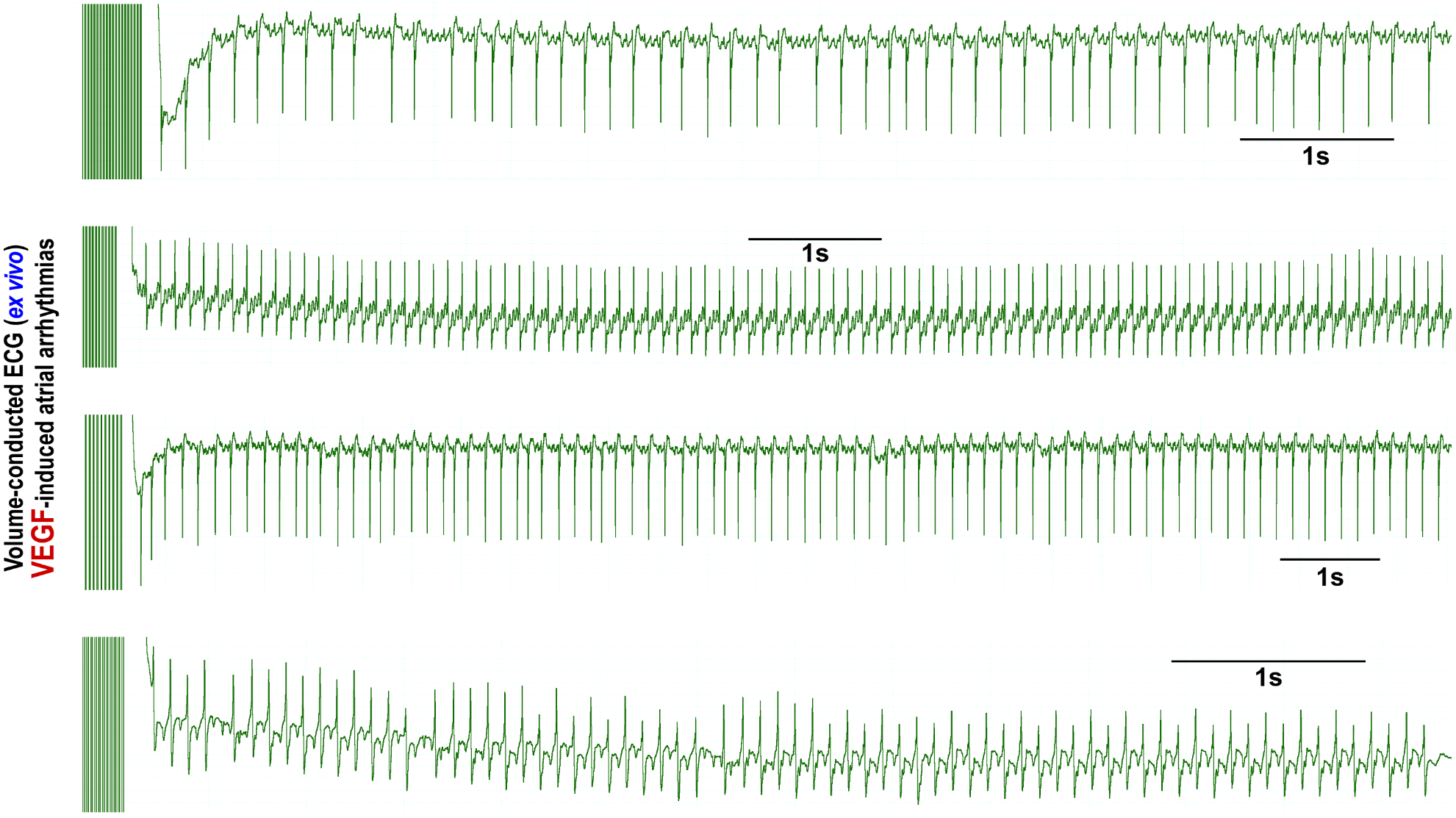
**

**Supplementary Figure 3. VEGF increases susceptibility to burst pacing-induced atrial arrhythmias.** Examples of atrial arrhythmias from VEGF (100 ng/l) –treated Langendorff-perfused hearts.

**SUPPLEMENTARY FIGURE 4**

**
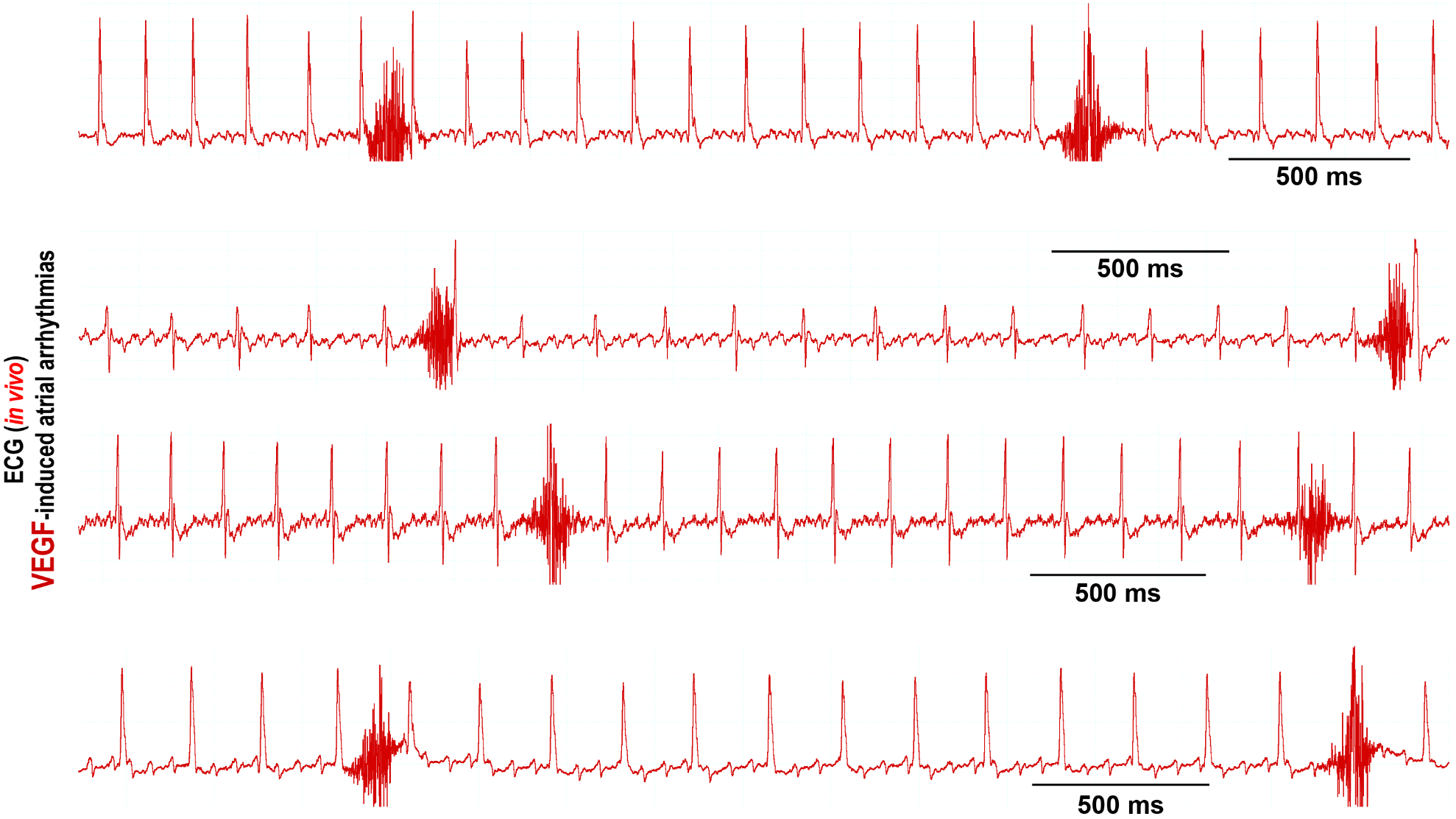
**

**Supplementary Figure 4. VEGF increases atrial arrhythmia susceptibility *in vivo*.** Examples of atrial arrhythmias elicited by caffeine + epinephrine challenge from VEGF (100 ng/l) –treated anesthetized mice.

**SUPPLEMENTARY FIGURE 5**

**
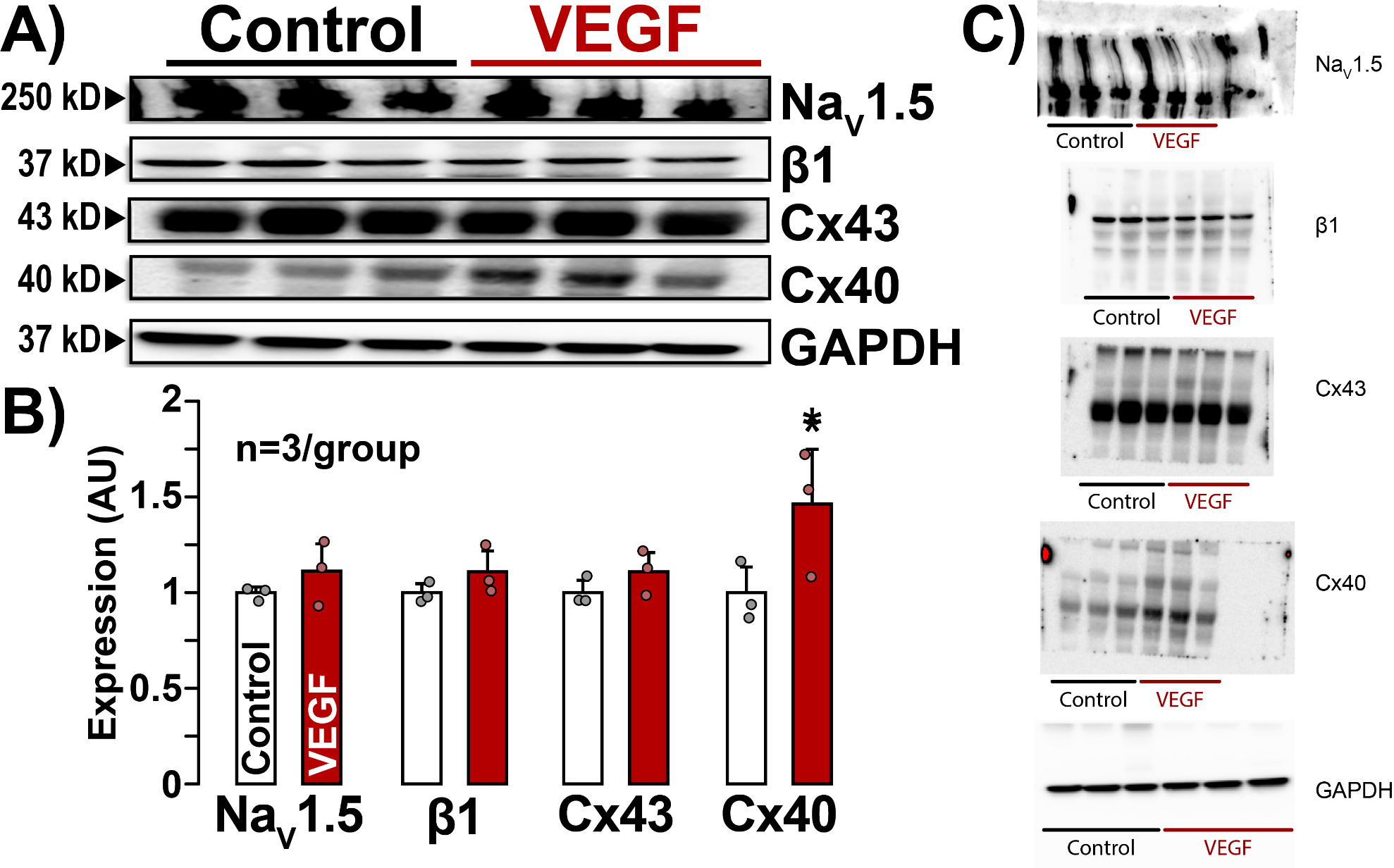
**

**Supplementary Figure 5. VEGF effects on expression of ID proteins.** **A)** Western immunoblots and **B)** summary quantification of ID protein expression from VEGF-treated and vehicle control hearts (n=3/group, * p<0.05 vs. control). All control and VEGF-treated samples were processed as one experiment on a single gel / membrane. **C)** Full-length blots.
